# The evolution of *Homo sapiens denisova *and *Homo sapiens neanderthalensis *miRNA targeting genes in the prenatal and postnatal brain

**DOI:** 10.1186/1471-2164-16-S13-S4

**Published:** 2015-12-16

**Authors:** Konstantin V Gunbin, Dmitry A Afonnikov, Nikolay A Kolchanov, Anatoly P Derevianko, Eugeny I Rogaev

**Affiliations:** 1 Center of Brain Neurobiology and Neurogenetics, Federal State Research Center Institute of Cytology and Genetics, Siberian Branch, Russian Academy of Sciences, Akademika Lavrenti'eva Ave. 10, Novosibirsk, 630090, Russia; 2 Novosibirsk State University, Pirogova St. 2, Novosibirsk, 630090, Russia; 3 National Research Centre Kurchatov Institute, Akademika Kurchatova pl. 1, Moscow, 123182, Russia; 4 Institute of Archaeology and Ethnography, Siberian Branch, Russian Academy of Sciences, Akademika Lavrent'eva Ave. 17, Novosibirsk, 630090, Russia; 5 Lomonosov Moscow State University, Faculty of Bioengineering and Bioinformatics, Moscow, Russia; 6 Vavilov Institute of General Genetics, Gubkina 3, Moscow, Russia; 7 BNRI, Department of Psychiatry, University of Massachusetts Medical School, 303 Belmont Street, Worcester, MA 15604, USA

**Keywords:** miRNA, functionally important substitutions, *Homo sapiens neanderthalensis*, *Homo sapiens denisova*

## Abstract

**Background:**

As the evolution of miRNA genes has been found to be one of the important factors in formation of the modern type of man, we performed a comparative analysis of the evolution of miRNA genes in two archaic hominines, *Homo sapiens neanderthalensis *and *Homo sapiens denisova*, and elucidated the expression of their target mRNAs in bain.

**Results:**

A comparative analysis of the genomes of primates, including species in the genus *Homo*, identified a group of miRNA genes having fixed substitutions with important implications for the evolution of *Homo sapiens neanderthalensis *and *Homo sapiens denisova*. The mRNAs targeted by miRNAs with mutations specific for *Homo sapiens denisova *exhibited enhanced expression during postnatal brain development in modern humans. By contrast, the expression of mRNAs targeted by miRNAs bearing variations specific for *Homo sapiens neanderthalensis *was shown to be enhanced in prenatal brain development.

**Conclusions:**

Our results highlight the importance of changes in miRNA gene sequences in the course of *Homo sapiens denisova *and *Homo sapiens neanderthalensis *evolution. The genetic alterations of miRNAs regulating the spatiotemporal expression of multiple genes in the prenatal and postnatal brain may contribute to the progressive evolution of brain function, which is consistent with the observations of fine technical and typological properties of tools and decorative items reported from archaeological Denisovan sites. The data also suggest that differential spatial-temporal regulation of gene products promoted by the subspecies-specific mutations in the miRNA genes might have occurred in the brains of *Homo sapiens denisova *and *Homo sapiens neanderthalensis*, potentially contributing to the cultural differences between these two archaic hominines.

## Background

Early in the 21^st ^century, fossils of an ancient man who lived 50-45 thousand years (ky) ago were found in the Denisova cave in the Altai mountains [1]. The *Homo sapiens denisova *(*H. s. d.*) nuclear genome was first sequenced in 2010 [2] and then re-sequenced in 2012 [3]. The *Homo sapiens neanderthalensis *(*H. s. n.*) nuclear genome was first sequenced in 2010 [4] and for a second time in 2014 [5]. A comparative analysis of the archaic hominine (*H. s. d., H. s. n.) *and *Homo sapiens sapiens *(*H. s. s.*) genomes assessed the genetic contribution of *H. s. d*. and *H. s. n *to the genetic and biological profile of the modern *H. s. s*. populations [6]. It was shown that the *H. s. n*. lineage is a sister lineage to *H. s. d*. [2]. The population split of Neanderthals and Denisovans from modern humans was estimated to have occurred 550-765 ky ago and the split time of Neanderthals and Denisovans 380-473 ky ago [5]. Considering that the evolution of miRNA genes could be one of the key factors in the formation of the modern type of man [7-10], we analyzed the evolution of miRNA genes in *H. s. n*. and *H. s. d*. and the expression of their target mRNAs. Using improved versions of the *H. s. d*. and *H. s. n*. genomes, we performed computer-assisted comparisons of the *H. s. n., H. s. d*. and *H. s. s*. genomes to reveal the structural and functional organization of microRNAs (miRNAs) as well as the mRNAs targeted by these miRNAs. In both the Neanderthal and Denisovan genomes, we found miRNA genes with fixed substitutions in mature miRNAs and multiple substitutions in pre-miRNA regions involved in pre-miRNA processing.

Our analysis of spatiotemporal gene expression in human tissues demonstrated that the miRNAs bearing new genetic variants fixed in the Denisovan genome regulated target mRNAs with the highest levels of expression in the postnatal human brain.

## Results and discussion

To identify miRNA genes that evolved divergently in the genus Homo, we first used the map and pre-miRNA gene sequence data for 1595 experimentally confirmed *H. s. s*. miRNAs from the miRBase database rel. 19 [11]. We selected the *H. s. d*. and *H. s. n*. pre-miRNAs with highly confident sequences that are orthologous to *H. s. s*. pre-miRNAs. Using the selection procedure described in the Methods section (Selection of miRNA genes in *H. s. d*. and *H. s. n*. with no match in *H. s. s*.), we identified 1298 *H. s. d*. and 1329 *H. s. n*. pre-miRNAs perfectly matched to *H. s. s*. pre-miRNA gene sequences. In addition, we identified and selected for further study 106 *H. s. d*. and 102 *H. s. n*. diverged genes for pre-miRNA sequences, which were different from *H. s. s*. pre-miRNA genes by at least a single nucleotide. The other 191 *H. s. d*. and 164 *H. s. n*. pre-miRNAs did not pass quality sequence control in the selection procedure

Further, we explored the alignment of the genomes of six primates available in Ensembl rel. 69 [12]. Each of the selected *H. s. d*. and *H. s. n*. pre-miRNA genes non-identical to *H. s. s*. orthologs had at least one ortholog in another primate. This result indicated that these pre-miRNA coding genes were present in the genome of the common ancestor of *H. s. s.*, *H. s. d*. and *H. s. n*. long before the evolutionary split between these hominines. In following up this analysis, we confirmed the existence of orthologs for all selected *H. s. d*. and *H. s. n*. miRNA genes except one *H. s. d*. miRNA gene.

Next, we analyzed all substitutions found in the archaic hominine miRNA genes selected as described above. First, we excluded a few doubtful nucleotide substitutions observed in the sequencing data for the pre-miRNA genes of *H. s. d*. and *H. s. n*. (see Methods section). Then, we mapped the remaining genetic variants found in the pre-miRNA genes of *H. s. d*. and *H. s. n*. onto the secondary structures of the corresponding pre-miRNAs in *H. s. s*. available at the miRBase rel. 19 [11]. We selected the *H. s. d*. and *H. s. n*. pre-miRNAs with (i) nucleotide substitutions (deletions, insertions) in the regions corresponding to the sequences of mature miRNAs (and/or miRNAs*) responsible for binding to target mRNAs or (ii) multiple (two or more) densely spaced substitutions within a pre-miRNA region involved in pre-miRNA processing. From these pre-miRNA groups, we excluded the pre-miRNAs that occur in the *H. s. s*. genome in more than one copy. The aim of this exclusion was to select pre-miRNAs with unique functions. The selection yielded *H. s. d*. and *H. s. n*. pre-miRNAs with nucleotide substitutions that might contribute to significant functional differences from the *H. s. s*. pre-miRNAs.

The functional annotation of the diverged pre-miRNA genes in archaic hominines was performed as follows. First, we selected the miRNAs expressed in the central nervous system (CNS) using the miRGator 3.0 [13] and ChIP-seq data for H3K4me3 (histone H3 trimethyl K4) modified histones marking active promoters in primate cortical neurons [14]. Second, the potential target mRNAs of these central nervous system (CNS)-active miRNAs were identified using data from miRGator 3.0 [13]. miRGator 3.0 contains experimental miRNA/target-mRNAs co-expression data obtained under various conditions. Therefore, we extracted from miRGator 3.0 genes with co-expression correlation coefficient with miRNA *r*≤-0.9 [13]. We used the co-expression evidence to identify putative interactions between miRNAs and their target mRNAs instead of the CLIP-seq experiments results because the latter technology requires cell lysis, which facilitates the interaction of components that are usually segregated by cellular compartments [15], and many miRNA/mRNAs interactions identified by CLIP-seq are non-canonical, which in turn does not mediate repression [16]. However, co-expression data indicate pathways (not genes) targeted by miRNA but do not allow the identification of direct miRNA/mRNA interactions. The latter has a side advantage because, in this approach, we selected against target genes with limited effect on tissue functioning [17]. Third, to identify the human brain structures (topologically different brain regions) showing increased levels of the target mRNAs (henceforth referred to as the target brain structures), we performed a randomization test using the Human Allen Brain Atlas [18] and the BrainSpan Atlas [19]. We employed the randomization test for this purpose because the standard analytic annotation enrichment techniques are inapplicable for miRNA functional enrichment analysis [20].

The characteristics of the nucleotide substitutions in each of the CNS-active pre-miRNA genes diverged in Denisovan and Neanderthal are shown in Tables 1 and 2. These miRNA genes were stratified into four groups. The first group consists of 9 miRNAs with fixed mutations unique to the *H. s. d*. lineage. The second group includes 5 miRNAs with fixed mutations unique to the *H. s. n*. lineage. Two other miRNA gene groups were compiled of 14 *H. s. d*. and 18 *H. s. n*. miRNA genes bearing variants that match to one of the polymorphic alleles in *H. s. s*. Most polymorphisms in these miRNAs are SNPs (single nucleotide polymorphisms). Several substitutions are observed in the seed region (based on Table 1 and 2) in six out of 14 *H. s. d*. and five out of 18 *H. s. n*. pre-miRNAs common to human polymorphisms. Only one indel in the seed region was observed in hsa-mir-3161 in both *H. s. n*. and *H. s. d*. The minority of miRNAs with polymorphisms in the seed region is not sufficient to claim that the general capacity of these miRNA pools have altered binding specificity to target mRNAs. This result allowed us to use the third and fourth pools as a control to compare the target specificities with miRNA bearing fixed variants specific for *H. s. n*. and *H. s. d*.

**Table 1:** *Homo sapiens denisova *miRNAs that differ from *Homo sapiens sapiens *miRNAs.

				Genome position (hg 19)					
							
#	miRNA*	Ancestral state (h: human, d: Denisovan)	Difference (human/ Denisovan)**	Chromosome	Position	Strand	DB SNP ID	Allele frequency **(1000 genomes **[28]**)**	African population frequency **(1000 genomes **[28]**or dbSNP 141 **[29]**)**
***Homo sapiens denisova *miRNA mutations common to previously observed human miRNA polymorphisms**									

1	**{hsa-mir-1178}**	d	***{A/G}***	12	120151493	-	rs7311975	C:0.1382	0.4191

2	**{hsa-mir-1252}**	d	**{A/G}**	12	79813049	+	rs115256251	G:0.0032	0.0098

3	**{hsa-mir-1269a}**	d	**{G/A}**	4	67142620	+	rs73239138	A: 0.3942	0.7277

4	{hsa-mir-146a}	d	***{C/G}***	5	159912418	+	rs2910164	C:0.3815	0.475(YRI)

5	**{hsa-mir-2682 (3p)}**	d	***{G/A}***	1	98510847	-	rs74904371	T:0.0208	0.0053

6	**{hsa-mir-3161}**	d	***{-/A}***	11	48118347	+	rs11382316	A:0.7599	+(BUSHMAN)

7	**{hsa-mir-4804 (5p)}**	d	***{C/G}***	5	72174432	+	rs266435	G: 0.8001	0.7269

8	**{hsa-mir-608}**	d	**{C/G}**	10	102734778	+	rs4919510	G:0.3638	0.4402

		h	A/G	10	102734813	+	-	-	-

9	**hsa-mir-3124 (3p)**	d	**C/A**	1	249120631	+	rs115160731	A:0.0128	0.0469

10	**hsa-mir-4514**	h	**T/C**	15	81289798	-	rs116034786	G:0.0136	0.0371

11	**hsa-mir-1269b**	h	***C/(G|C)***	17	12820646	-	rs7210937	C:0.3520	0.5976

12	hsa-mir-6085	h	**A/-**	15	62635322	+	rs372168584	-:0.0006	0.0023

13	**hsa-mir-378e**	d	T/C	5	169455502	+	rs367764573	C:0.0026	0.0008

		h	***T/C***	5	169455551	+	rs376752141	C:0.0026	0.0008

14	hsa-mir-662	h	T/A	16	820215	+	rs74656628	A:0.1374	0.3971

		h	***G/A***	16	820249	+	rs9745376	A:0.0543	0.1921

15	hsa-mir-4463	d	-/AG	6	76138146	+	rs5877455	AG:0.7079	0.9198

16	**{hsa-mir-532** **(3p, 5p)}**	d	{A/G}	X	49767832	+	rs456615	G:1	0.7587

17		d	{A/G}	X	49767835	+	rs456617	G:1	0.7587

18	**{hsa-mir-943}**	h	A/G	4	1988176	-	rs368905227	T: 0.0004	0

		d	{-/AG}	4	1988188	-	rs3034718	CT: 0.2766	0.4629

		d	{A/G}	4	1988193	-	rs1077020	C: 0.2504	0.3865

*Homo sapiens denisova*-specific mutations									

1	**hsa-mir-1321**	h	***G/T***	X	85090839	+	-	-	

2	hsa-mir-1909	h	**G/C**	19	1816168	-	-	-	

3	**hsa-mir-3143**	h	**T/C**	6	27115417	+	-	-	

4	**hsa-mir-3152 (5p)**	h	**A/-**	9	18573332	+	-	-	

5	hsa-mir-3185	h	**G/A**	17	46801808	-	-	-	

6	**hsa-mir-4478**	h	***T/A***	9	124882437	-	-	-	

7	**hsa-mir-4700 (3p)**	h	**C/T**	12	121161049	+	-	-	

8	**hsa-mir-4710**	h	**T/C**	14	105144064	-	-	-	

9	**hsa-mir-5687**	h	***C/T***	5	54804702	-	rs545080149	A:0.0002	0.0008

10	**hsa-mir-609**	h	***G/A***	10	105978625	-	-	-	

11	**{hsa-mir-671 (3p)}**	h	**{C/T}**	7	150935593	+	-	-	

* bold - expression in the central neural system (miRGator 3.0 data [13] and data from [14]), underline - common between *H. s. d*. and *H. s. n*.

** bold - location in mature miRNA, {} common between *H. s. d*. and *H. s. n*., italic - location in canonical seed (nucleotides 2-7) region, based on [40]

**Table 2:** *Homo sapiens neanderthalensis *miRNAs that differ from *Homo sapiens sapiens *miRNAs.

				Genome position (hg 19)					
							
#	miRNA*	Ancestral state (h: human, n: Neanderthal; c: chimpanzee)	Mutation (human/ Neanderthal)**	Chromosome	Position	Strand	DB SNP ID	Allele frequency **(1000 genomes **[28]**)**	African population frequency **(1000 genomes **[28]**or dbSNP 141 **[29]**)**
*Homo sapiens neanderthalensis *miRNA mutations common to previously observed human miRNA polymorphisms									

1	**{hsa-mir-1178}**	n	***{A/G}***	12	120151493	-	rs7311975	C:0.1382	0.4191

2	**{hsa-mir-1252}**	n	**{A/G}**	12	79813049	+	rs115256251	G:0.0032	0.0098

3	**{hsa-mir-1269a}**	n	**{G/A}**	4	67142620	+	rs73239138	A:0.3942	0.7277

4	{hsa-mir-146a}	n	***{C/G}***	5	159912418	+	rs2910164	C:0.3815	0.475(YRI)

5	**{hsa-mir-2682 (3p)}**	n	***{G/A}***	1	98510847	-	rs74904371	T:0.0208	0.0053

6	**{hsa-mir-3161}**	n	***{-/A}***	11	48118347	+	rs11382316	A:0.7599	+(BUSHMAN)

7	**{hsa-mir-4804 (5p)}**	n	***{C/G}***	5	72174432	+	rs266435	G:0.8001	0.7269

8	**{hsa-mir-608}**	n	**{C/G}**	10	102734778	+	rs4919510	G:0.3638	0.4402

9	**hsa-mir-1343**	n	**T/C**	11	34963416	+	rs2986407	C:0.7668	0.792

10	**hsa-mir-3129 (5p)**	h	**G/C**	2	189997816	-	rs192364638	C:1	1

11	**hsa-mir-4274**	n	**-/CAC**	4	7461827	+	rs35245133	CAC:0.858	+(BUSHMAN)

12	**hsa-mir-4293**	h	***C/G***	10	14425221	-	rs12220909	G:0.9619	0.9992

13	hsa-mir-5189	n	G/C	16	88535400	+	rs80296158	C:0.0507	0.1831

14	**hsa-mir-149** **(3p, 5p)**	n	A/G	2	241395500	+	rs71428439	G:0.1440	0.1112

15		n	T/C	2	241395503	+	rs2292832	C:0.3866	0.2716

16	**hsa-mir-1908**	n	A/G	11	61582708	-	rs174561	C:0.2796	0.0197

		h	G/A	11	61582709	-	-	-	-

17	**hsa-mir-3938**	n	TT/-	3	55886573	-	rs10575780	-:0.2426	0.4667

18	hsa-mir-4463	n	-/AG	6	76138146	+	rs398110299	AG:0.7079	0.9198

19	hsa-mir-4719	n	T/C	16	76902847	+	rs7500280	C:0.3293	0,2171

		n	G/A	16	76902850	+	rs7499278	A:0.7528	0.9455

20	**{hsa-mir-532}** **{(3p, 5p)}**	n	{A/G}	X	49767832	+	rs456615	G:1	0.7587

21		n	{A/G}	X	49767835	+	rs456617	G:1	0.7587

22	**{hsa-mir-943}**	n	{-/GA}	4	1988188	-	rs3034718	CT:0.2766	0.4629

		n	{A/G}	4	1988193	-	rs1077020	C:0.2504	0.3865

*Homo sapiens neanderthalensis-*specific mutations									

1	**hsa-mir-1208**	h	***T/C***	8	129162377	+	-	-	

2	hsa-mir-4532	h	***C/T***	20	56470458	+	-	-	

3	**hsa-mir-4718**	h	**C/T**	16	12814185	+	-	-	

4	hsa-mir-615	h	**C/T**	12	54427811	+	-	-	

5	**hsa-mir-639**	h	***T/C***	19	14640435	+	rs561305115	C:0.0064	0.004

		h	G/C	19	14640399	+	rs372602559	C:0.0062	0.004

6	**{hsa-mir-671 (3p)}**	h	**{C/T}**	7	150935593	+	-	-	

7	hsa-mir-6715b (3p)	h	**G/T**	10	114059393	-	rs182337914	A:0.0012	0

8	hsa-mir-4749 (3p)	h	***C/(C|T)***	19	50357891	+	rs372882504	-	1(C)

9	**hsa-mir-3939**	h(A)/c(G)	A/(G|A)	6	167411298	-	rs80032204	-	0.915(A)

		h(A)/c(G)	A/(G|A)	6	167411300	-	rs77840042	-	0.912(A)

		h(C)/c(T)	C/(C|T)	6	167411301	-	rs75692943	-	0.912(C)

		h	***G/(G|A)***	6	167411334	-	rs75823810	-	0.954(G)

		h	***C/(C|T)***	6	167411337	-	rs73024232	-	0.957(C)

		h	C/(C|T)	6	167411362	-	rs77072520	-	0.972(C)


* bold - expression in the central neural system (MiRGator 3.0 data [13] and data from [14])

** bold - location in mature miRNA, {} - common between *H. s. d*. and for *H. s. n.*, italic - location in canonical seed (nucleotides 2-7) region, based on [40]

Next, we searched for the brain regions and CNS development stage showing the most abundant expression of mRNAs targeted by the miRNAs that diverged in archaic hominines (see Methods section). For this purpose, we used data in (1) the BrainSpan Exon microarray (BrainSpan) [19] and (2) the Allen Human Brain Atlas as updated March 7, 2013 (AHBA) [18].

**Figure 1:**
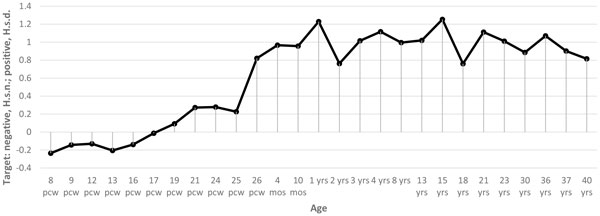
**Target stages of human brain development for *H. s. d*. and *H. s. n*. miRNA pools expressed in neural tissues and having unique mutations**. *Y*-axis, comparisons of Cohen's *d *values of the regulatory potential for selected *H. s. d*. (positive values) and *H. s. n*. (negative values) miRNA pools; *X*- axis, age: pcw - post conception weeks, m - postnatal months, y - postnatal years.

**Figure 2:**
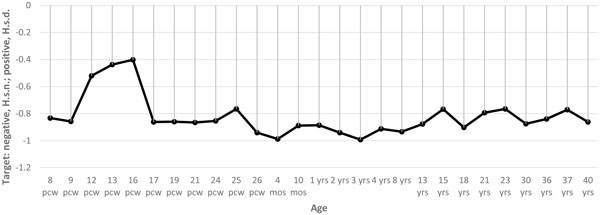
**Target stages of human brain development for *H. s. d*. and *H. s. n*. miRNA pools expressed in neural tissues and having mutations shared with known human polymorphisms**. *Y*-axis, comparisons of Cohen's *d *values of the regulatory potential for selected *H. s. d*. (positive values) and *H. s. n*. (negative values) miRNA pools; *X*-axis, age: pcw - post conception weeks, m - postnatal months, y - postnatal years.

**Figure 3:**
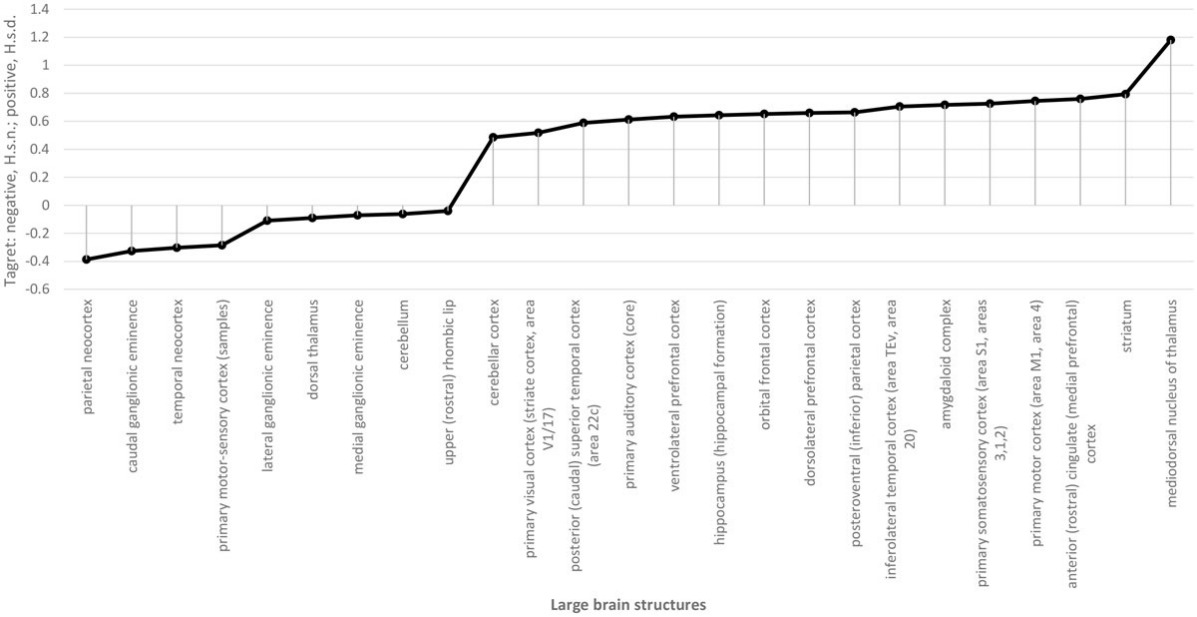
**Target structures of human brain for tested and control *H. s. d*. and *H. s. n*. miRNA pools expressed in neural tissues and having unique mutations**. *Y*-axis, comparisons of Cohen's *d *values of the regulatory potential for selected *H. s. d*. (positive values) and *H. s. n*. (negative values) miRNA pools; *X*- axis, large brain structures.

The results of the analysis of BrainSpan data [19] demonstrated that mRNAs targeted by miRNAs bearing fixed mutations specific for *H. s. d*. are enriched in the postnatal stage of brain development, whereas mRNAs targeted by miRNAs bearing fixed mutations specific for *H. s. n*. are most abundant in the prenatal brain (Figure 1). In contrast, no development stage-dependent expression was found for mRNAs targeted by miRNAs matched to polymorphic variants in *H. s. s*. (Figure 2). Thus, we can anticipate the differential effect of genetic variations found in archaic hominines on brain function and development in Denisovans and Neanderthals. The effects are not likely to be due to bias in the number of mRNA targets. The number of targeted genes is similar between miRNAs bearing fixed mutations specific for *H. s. n*. and *H. s. d*. (Ensembl gene IDs): 1260 target genes for *H. s. n*. and 1547 for *H. s. d*. (excluding targets of hsa-mir-671-3p miRNA that possess specific variants common for *H. s. d*. and *H. s. n*.). In addition, the number of targeted genes is similar between *H. s. n*. and *H. s. d*. miRNAs matched to polymorphic variants in *H. s. s*. (Ensembl gene IDs): 4072 target genes for *H. s. n*. and 3653 for *H. s. d*. It is interesting that the highest expression of transcripts targeted by miRNAs with polymorphisms unique for *H. s. d*. is confined to the broad thalamus regions and the prefrontal cortex (Figure 3). Nevertheless, there are multiple forebrain regions known to be important for speech that exhibit higher expression of miRNA targets from the set with polymorphisms unique for *H. s. d*. (Figure 3): the inferiolateral temporal cortex, medial and ventrolateral prefrontal cortex. It is of interest that the thalamus plays a significant role in the shaping of the human language-ready brain [21].

**Figure 4:**
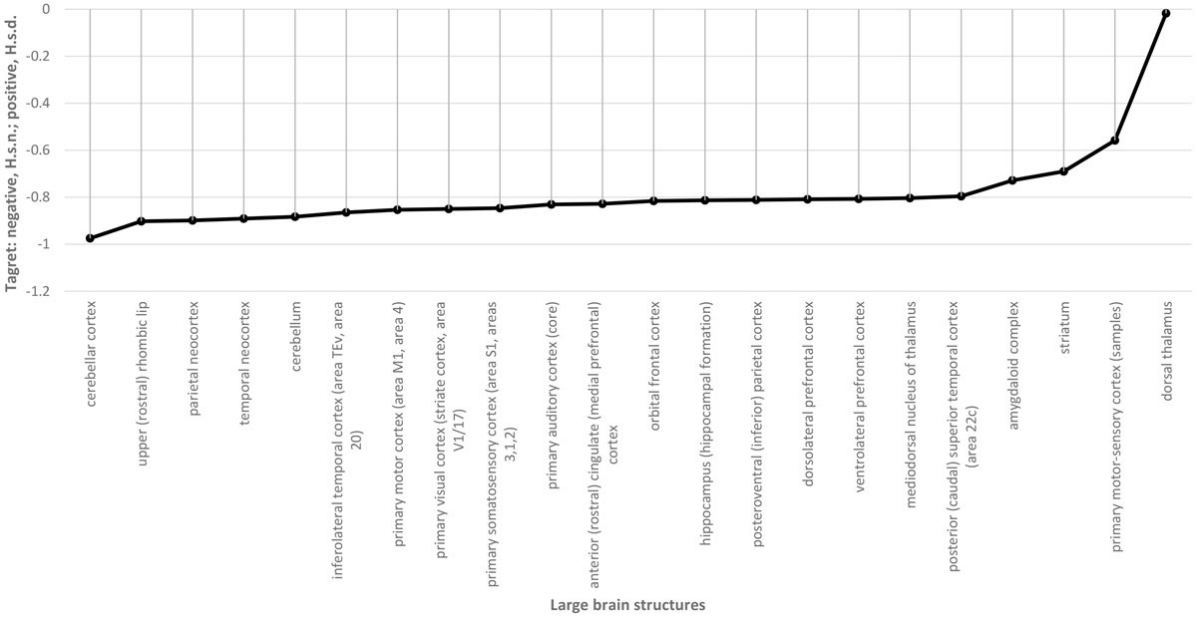
**Target structures of human brain for tested and control *H. s. d*. and *H. s. n*. miRNA pools expressed in neural tissues and having mutations shared with known human polymorphisms**. *Y*-axis, comparisons of Cohen's *d *values of the regulatory potential for selected *H. s. d*. (positive values) and *H. s. n*. (negative values) miRNA pools; *X*- axis, large brain structures.

In contrast, when we consider miRNAs of ancient humans expressed in the central nervous system and having variants shared with known human polymorphisms, the picture changes drastically (Figure 4): all brain structures are targeted more by polymorphic *H. s. n*. miRNAs than by polymorphic *H. s. d*. miRNAs. The larger signal in *H. s. n.*, shown in Figures 2 and 4, is likely due to a higher number of miRNAs expressed in the central nervous system and sharing variation with the human population for *H. s. n*. (18, see Table 2) than for *H. s. d*. (14, see Table 1). Considering common miRNAs sharing human polymorphisms in these organisms (10 miRNAs), the specific numbers are 4 and 8, respectively (351 *H. s. d*.-specific targets versus 972 *H. s. n*.-specific targets (Ensembl gene IDs)). Given that the largest number of gene targets was found for the polymorphic hsa-mir-149 (5p) miRNA (Table 3) in *H. s. n.*, this difference between the two hominine subspecies can be attributed to this particular miRNA.

**Table 3:** Number of target genes for each miRNA shown in Tables 1 and 2, based on MiRGator 3.0 data [13].

miRNA	Number of target genes (Ensembl gene IDs)*
hsa-mir-1178	701

hsa-mir-1208	878

hsa-mir-1252	1228

hsa-mir-1269a	639

hsa-mir-1269b	43

hsa-mir-1321	1159

hsa-mir-1343	128

hsa-mir-146a	140

hsa-mir-149 (3p)	181

hsa-mir-149 (5p)	495

hsa-mir-1908	25

hsa-mir-1909	33

hsa-mir-2682 (3p)	15

hsa-mir-3124 (3p)	151

hsa-mir-3129 (5p)	101

hsa-mir-3143	121

hsa-mir-3152 (5p)	33

hsa-mir-3161	62

hsa-mir-3185	102

hsa-mir-378e	49

hsa-mir-3938	65

hsa-mir-3939	1

hsa-mir-4274	42

hsa-mir-4293	0

hsa-mir-4463	40

hsa-mir-4478	76

hsa-mir-4514	134

hsa-mir-4532	10

hsa-mir-4700 (3p)	76

hsa-mir-4710	52

hsa-mir-4718	42

hsa-mir-4719	73

hsa-mir-4749 (3p)	32

hsa-mir-4804 (5p)	1

hsa-mir-5189	0

hsa-mir-532 (3p)	598

hsa-mir-532 (5p)	454

hsa-mir-5687	0

hsa-mir-608	1039

hsa-mir-6085	-**

hsa-mir-609	538

hsa-mir-615	200

hsa-mir-639	625

hsa-mir-662	256

hsa-mir-671 (3p)	168

hsa-mir-6715b (3p)	-**

hsa-mir-943	512

* genes with Pearson correlation coefficient of co-expression ≤-0.9.

** these miRNAs were not annotated in mirGator 3.0

The fine target structures identified using AHBA data for gene expression in the left hemisphere of the brain [18] are presented in Tables 4 and 5. For identification of the fine target brain structures, we used the difference in regulatory potential between the *H. s. d*. and *H. s. n*. samples (Table 4, 5) as a marker (see Methods section). Importantly (Table 4), the difference between the target structures of unique *H. s. d*. and *H. s. n*. miRNAs expressed in the central nervous system tissues is not significant (effect size measured as the absolute value of difference between Cohen's *d *statistics<0.5) for all fine brain structures except two: the fusiform gyrus for *H. s. d*. miRNAs and the precentral gyrus for *H. s. n*. miRNAs. The picture obtained based on the target mRNAs for miRNAs expressed in the central nervous system tissues with known human polymorphisms (Table 4) is the same (compare Table 4 and 5): for the vast majority of fine brain structures, the difference between targeting by *H. s. d*. and *H. s. n*. is not significant, except for two thalamus structures and the lateral orbital gyrus, targeted by *H. s. d*. miRNAs. It is important that the fusiform and lateral orbital gyri are responsible for face perception and socialization, respectively [22,23]. Taken together, these findings indicate an important role of unique *H. s. d*. miRNAs in the development of specific brain structures in Denisovans, which likely enabled them to reach an unparalleled level of craftsmanship [1].

**Table 4:** Results of the identification of brain structures of the left hemisphere characterized by the difference in regulatory potential for *H.s. d*. and *H. s. n*. miRNAs expressed in neural tissues and having unique substitutions.

AHBA Struct. ID *	Structure description. ID **	*p*, *H. s. d*.	*p*, *H. s. n*.	Cohen's *d*, *H. s. d*.	Cohen's *d*, *H. s. n*.	(Cohen's *d, H. s. d*.) - (Cohen's *d, H. s. n*.)
4158	fusiform g., b. of the its	0.00226	0.00662	5.94882	5.26565	0.68317

4273	short insular gyri	0.0041	0.0062	5.65202	5.40279	0.24923

4045	inferior frontal g., orbital pt.	0.00676	0.00884	5.37587	5.16541	0.21046

4023	superior frontal g., medial b.	0.00552	0.00626	5.46649	5.30947	0.15702

4090	postcentral g., b. of the posterior central sulcus	0.00764	0.00988	5.21272	5.07402	0.1387

4120	precuneus, superior lateral b.	0.00702	0.00788	5.30175	5.16909	0.13266

4060	lateral orbital g.	0.00804	0.00972	5.19963	5.07262	0.12701

4143	middle temporal g., inferior b.	0.00816	0.00936	5.1509	5.04074	0.11016

4088	postcentral g., superior lateral aspect	0.00856	0.00978	5.15144	5.04957	0.10187

4039	inferior frontal g., triangular pt.	0.00786	0.00906	5.20687	5.11107	0.0958

4215	superior occipital g., inferior b.	0.0076	0.00796	5.23815	5.16956	0.06859

4251	subiculum	0.00644	0.0066	5.35415	5.28977	0.06438

4074	paracentral lobule, anterior pt., inferior b.	0.00912	0.0091	5.15364	5.09767	0.05597

4223	cingulate g., frontal pt., superior b.	0.00404	0.0042	5.62605	5.58632	0.03973

4288	putamen	0.00728	0.00834	5.23485	5.19766	0.03719

4160	fusiform g., b. of cos	0.00712	0.00692	5.30752	5.27444	0.03308

4245	parahippocampal g., b. of cos	0.00762	0.00692	5.24375	5.22006	0.02369

4014	precentral g., inferior lateral aspect	0.0085	0.00936	5.11266	5.09452	0.01814

4136	superior temporal g., inferior b.	0.00764	0.00728	5.20265	5.23345	-0.0308

4051	medial orbital g.	0.0067	0.00554	5.35583	5.43806	-0.08223

4224	cingulate g., frontal pt., inferior b.	0.00664	0.00608	5.26935	5.35976	-0.09041

4087	postcentral g., b. of the central sulcus	0.0096	0.00756	5.12513	5.23082	-0.10569

4098	supraparietal lobule, superior b.	0.00742	0.00572	5.23545	5.3476	-0.11215

4151	inferior temporal g., b. of mts	0.00888	0.00732	5.11307	5.22869	-0.11562

4030	middle frontal g., superior b.	0.00984	0.00766	5.08151	5.20719	-0.12568

4201	occipito-temporal g., inferior b.	0.00948	0.00782	5.05788	5.20134	-0.14346

4031	middle frontal g., inferior b.	0.00548	0.00426	5.39941	5.54788	-0.14847

4024	superior frontal g., lateral b.	0.00308	0.00302	5.72827	5.89251	-0.16424

4142	middle temporal g., superior b.	0.00948	0.00736	5.08104	5.25797	-0.17693

4150	inferior temporal g., lateral b.	0.00704	0.0054	5.30094	5.50188	-0.20094

4149	inferior temporal g., b. of the its.	0.0095	0.00712	5.09672	5.32253	-0.22581

4159	fusiform g., lateral b.	0.00944	0.00742	5.03024	5.25885	-0.22861

4214	superior occipital g., superior b.	0.00612	0.0047	5.31198	5.55676	-0.24478

4258	dentate g.	0.0073	0.00506	5.29347	5.56439	-0.27092

4193	lingual g., peristriate	0.00976	0.00616	5.08997	5.4184	-0.32843

4013	precentral g., superior lateral aspect	0.00968	0.00596	5.05046	5.38028	-0.32982

4015	precentral g., b. of the central sulcus	0.00898	0.00342	5.10895	5.70299	-0.59404

* - For each target brain structure, the following data are provided: (1) the AHBA ID and (2) its description; (3) and (4) *p*, the probability of observing $L_{krand}\geq L_{krand}^{Q}$ by random chance using unique *H. s. d*. and *H. s. n*. miRNAs expressed in central nervous system tissues; (5) and (6) the Cohen's *d *statistic of difference between *L_krand _*and $L_{krand}^{Q}$ value distributions for unique *H. s. d*. and *H. s. n*. miRNAs expressed in the central nervous system tissues; (7) difference between these Cohen's *d *statistics.

** - g., gyrus; b., bank; pt., part.

**Table 5:** Results of the identification of brain structures of the left hemisphere characterized by the difference between regulatory potential for *H.s. d*. and *H. s. n*. miRNAs expressed in neural tissues and having substitutions shared with known human polymorphisms.

AHBA Struct. ID *	Structure description. ID **	*p*, *H. s. d*.	*p*, *H. s. n*.	Cohen's *d*, *H. s. d*.	Cohen's *d*, *H. s. n*.	(Cohen's *d, H. s. d*.) - (Cohen's *d, H. s. n*.)
4060	lateral orbital g.	0.00194	0.00816	5.92943	5.18602	0.74341

4288	putamen	0.0018	0.00634	6.03981	5.29693	0.74288

4258	dentate g.	0.00156	0.0045	6.18038	5.55627	0.62411

4901	inferior rostral g.	0.0045	0.0084	5.59337	5.12814	0.46523

4193	lingual g., peristriate	0.00268	0.00618	5.78555	5.32347	0.46208

4187	cuneus, striate	0.00474	0.00956	5.50591	5.04395	0.46196

4151	inferior temporal g., b. of mts	0.0042	0.00632	5.60432	5.29836	0.30596

4194	lingual g., striate	0.00714	0.00878	5.28597	5.12021	0.16576

4160	fusiform g., b. of cos	0.00622	0.00794	5.32299	5.17916	0.14383

4417	lateral group of nuclei, ventral division	0.00708	0.00514	5.27528	5.39697	-0.12169

4256	CA3 field	0.00958	0.00798	5.06621	5.22156	-0.15535

4169	transverse gyri	0.00936	0.00686	5.12785	5.29476	-0.16691

4282	body of caudate nucleus	0.00672	0.00534	5.25194	5.42113	-0.16919

4012	precentral g., b. of the precentral sulcus	0.0064	0.00446	5.29517	5.51095	-0.21578

4244	parahippocampal g., lateral b.	0.00688	0.00454	5.31264	5.53652	-0.22388

4186	cuneus, peristriate	0.00386	0.00274	5.64365	5.88129	-0.23764

4150	inferior temporal g., lateral b.	0.00776	0.00522	5.21501	5.45297	-0.23796

4106	supramarginal g., superior b.	0.00518	0.00342	5.46435	5.70277	-0.23842

4088	postcentral g., superior lateral aspect	0.0024	0.0015	5.93668	6.17562	-0.23894

4121	precuneus, inferior lateral b.	0.004	0.0023	5.65682	5.89645	-0.23963

4166	Heschl's g.	0.00858	0.00516	5.19857	5.44275	-0.24418

4013	precentral g., superior lateral aspect	0.00452	0.00298	5.55536	5.80945	-0.25409

4015	precentral g., b. of the central sulcus	0.00814	0.00564	5.16805	5.42259	-0.25454

4120	precuneus, superior lateral b.	0.00246	0.00182	5.89955	6.15638	-0.25683

4245	parahippocampal g., b. of the cos	0.00672	0.00426	5.31989	5.58364	-0.26375

4223	cingulate g., frontal pt., superior b.	0.0052	0.00352	5.41984	5.68604	-0.2662

4048	g. rectus	0.00998	0.00646	5.01861	5.28996	-0.27135

4074	paracentral lobule, anterior pt., inferior b.	0.0042	0.00242	5.61742	5.89182	-0.2744

4113	angular g., superior b.	0.00912	0.00522	5.10417	5.38524	-0.28107

4280	head of caudate nucleus	0.00482	0.00302	5.49717	5.78461	-0.28744

4114	angular g., inferior b.	0.00686	0.00394	5.33423	5.6244	-0.29017

4087	postcentral g., b. of the central sulcus	0.00414	0.00222	5.64523	5.93594	-0.29071

4030	middle frontal g., superior b.	0.0032	0.00166	5.79657	6.08899	-0.29242

4270	long insular gyri	0.0097	0.00618	5.07045	5.36942	-0.29897

4014	precentral g., inferior lateral aspect	0.00318	0.00174	5.80203	6.10501	-0.30298

4200	occipito-temporal g., superior b.	0.00542	0.00314	5.44127	5.74428	-0.30301

4255	CA2 field	0.00338	0.0016	5.78178	6.08596	-0.30418

4273	short insular gyri	0.00502	0.0029	5.47924	5.79333	-0.31409

4099	supraparietal lobule, inferior b.	0.00482	0.00234	5.56715	5.88285	-0.3157

4045	inferior frontal g., orbital pt.	0.00272	0.00138	5.81445	6.1365	-0.32205

4039	Inferior frontal g., triangular pt.	0.00808	0.00528	5.16319	5.49488	-0.33169

4230	cingulate g., parietal pt., superior b.	0.00918	0.00546	5.13338	5.46665	-0.33327

4159	fusiform g., lateral b.	0.00336	0.00222	5.69632	6.03013	-0.33381

4023	superior frontal g., medial b.	0.00146	0.0007	6.19956	6.53647	-0.33691

4136	superior temporal g., inferior b.	0.00402	0.0018	5.66885	6.0067	-0.33785

4031	middle frontal g., inferior b.	0.00106	0.00058	6.29966	6.63933	-0.33967

4208	inferior occipital g., inferior b.	0.0017	0.00084	6.10349	6.44887	-0.34538

4251	subiculum	0.00486	0.00248	5.51224	5.8579	-0.34566

4073	paracentral lobule, anterior pt., superior b.	0.00522	0.0031	5.46017	5.81365	-0.35348

4079	frontal operculum	0.00502	0.0028	5.49085	5.84766	-0.35681

4149	inferior temporal g., b. of the its	0.00308	0.00176	5.82221	6.1795	-0.35729

4257	CA4 field	0.00612	0.00322	5.37575	5.73712	-0.36137

4215	superior occipital g., inferior b.	0.00876	0.00438	5.16436	5.5294	-0.36504

4063	subcallosal g.	0.00762	0.00424	5.24311	5.61246	-0.36935

4214	superior occipital g., superior b.	0.0035	0.00216	5.64522	6.01907	-0.37385

4143	middle temporal g., inferior b.	0.00444	0.00228	5.57522	5.95362	-0.3784

4207	inferior occipital g., superior b.	0.00632	0.00364	5.26928	5.65709	-0.38781

4051	medial orbital g.	0.00214	0.00096	5.9903	6.39314	-0.40284

4898	superior rostral g.	0.00612	0.00272	5.39109	5.80014	-0.40905

4098	supraparietal lobule, superior b.	0.00442	0.00198	5.63525	6.04788	-0.41263

4089	postcentral g., inferior lateral aspect	0.00762	0.00398	5.25268	5.67386	-0.42118

4107	supramarginal g., inferior b.	0.00668	0.00342	5.34353	5.7746	-0.43107

4178	planum polare	0.0065	0.00232	5.33903	5.77072	-0.43169

4224	cingulate g., frontal pt., inferior b.	0.00554	0.00254	5.42489	5.86867	-0.44378

4090	postcentral g., b. of the posterior central sulcus	0.00486	0.00206	5.53829	5.99888	-0.46059

4024	superior frontal g., lateral b.	0.00074	0.00022	6.59581	7.06305	-0.46724

4158	fusiform g., b. of the its	0.00142	0.00038	6.22982	6.69803	-0.46821

4201	occipito-temporal g., inferior b.	0.00284	0.00124	5.76999	6.2488	-0.47881

4142	middle temporal g., superior b.	0.00698	0.00336	5.26624	5.76073	-0.49449

* - For each target brain structure, the following data are provided: (1) the AHBA ID and (2) its description; (3) and (4) *p*, the probability of observing $L_{krand}\geq L_{krand}^{Q}$ by random chance using *H. s. d*. and *H. s. n*. miRNA with known human polymorphisms expressed in central nervous system tissues; (5) and (6) the Cohen's *d *statistic of the difference between the *L_krand _*and $L_{krand}^{Q}$ value distributions for *H. s. d*. and *H. s. n*. miRNAs with known human polymorphisms expressed in central nervous system tissues; (7) the difference between these Cohen's *d *statistics.

** - g., gyrus; b., bank; pt., part.

In addition to the identification of target brain structures for *H. s. d*. and *H. s. n*. miRNA activity, we found highly enriched functional categories of target gene annotations. We used the DAVID 6.7 functional annotation system [24] and the SP_PIR_KEYWORDS data class, with genes expressed in the human brain as background (16767 genes from BrainSpan). The results of the functional enrichment test are shown in Table 6. The most important difference between the target genes for unique *H. s. d*. and *H. s. n*. miRNAs expressed in the central nervous system tissues is the transport category enriched in target genes of *H. s. d*. miRNAs. Similar observations can be made considering miRNAs expressed in central nervous system tissues with substitutions shared with known human polymorphisms: "functional response" categories (such as activator, transcription regulation, synapse) enriched in target genes of *H. s. d*. and the developmental category enriched in target genes of such *H. s. n*. miRNAs. It would be reasonable to speculate that that the evolutionary changes in miRNA-transregulators might contribute to alterations in functional activities in specific brain regions in Denisovans, whereas in Neanderthals, the mutations in miRNAs could promote alterations in brain development and structure.

**Table 6:** Annotation enrichment of genes targeted by *H. s. d*. and *H. s. n*. miRNAs expressed in neural tissues, based on DAVID 6.7 [24]; BrainSpan gene list (16767 genes expressed in human brain) used as a background.

SP_PIR_KEYWORDS category *	# of genes	Corrected *p*-value	
		
		Bonferroni	Benjamini
Target genes of unique *H. s. d*. miRNAs			

transport	176	0.04072	0.04072

Target genes of unique *H. s. n*. miRNAs			

**-**			

Target genes of *H. s. d*. miRNAs having substitutions shared with known human polymorphisms			

**phosphoprotein**	1018	1.56E-22	1.56E-22

**alternative splicing**	1013	4.70E-19	2.35E-19

transcription	298	3.72E-06	1.24E-06

transcription regulation	292	3.86E-06	9.64E-07

metal-binding	403	2.56E-05	5.13E-06

chromosomal rearrangement	63	7.14E-05	1.19E-05

zinc	302	8.79E-05	1.26E-05

activator	98	2.27E-04	2.84E-05

cell junction	76	0.00179	1.99E-04

zinc-finger	231	0.00455	4.56E-04

synapse	45	0.01100	0.00100

DNA-binding	246	0.02287	0.00193

Target genes of *H. s. n*. miRNAs having substitutions shared with known human polymorphisms			

**phosphoprotein**	565	3.62E-04	3.62E-04

**alternative splicing**	574	0.00226	0.00113

developmental protein	84	0.00665	0.00222

cytoplasm	279	0.00715	0.00179

* bold - common between target genes of *H. s. n*. and *H. s. d*. miRNAs having substitutions shared with known human polymorphisms.

## Conclusions

In this work, we identified miRNA genes of archaic humans bearing sequence variations in comparison with the modern human genome sequence: 29 genes for *H. s. d*. and 31 genes for *H. s. n*. Almost one-third of those genes contain variations specific for archaic humans (11 genes for *H. s. d*. and 9 for *H. s. n*.). The analysis of human gene expression data resulted in 9 *H. s. d*. and 5 *H. s. n*. genes with specific archaic variations and the expression of their human orthologs in the central nervous system.

The detailed analysis of the human brain gene expression data demonstrated that the brain regions with the most abundant expression of mRNAs targeted by the *H. s. d*. miRNAs with fixed mutations are confined to the thalamus and the prefrontal cortex (especially the fusiform gyrus), no large brain regions are targeted by *H. s. n*. miRNAs with specific mutations. The only small brain regions targeted by *H. s. n*. miRNAs with such mutations are the superior lateral aspect and the central sulcus of the precentral gyrus.

We identified differences in gene expression pattern during human brain development for the targets of human orthologs for the selected miRNAs. Targets for miRNA genes with mutations specific for *H. s. d*. were expressed predominantly in the later prenatal and early postnatal development stages. Targets for miRNAs with mutations specific for *H. s. n*. were expressed predominantly in early prenatal brain development stages. These results may reflect a potential association between the changes in the Denisovan miRNA genes reported in this study and the brain development of *H. s. d*., which likely allowed them to reach their high level of craftsmanship.

## Methods

### Selection of miRNA genes in *H. s. d*. and *H. s. n*. with no match in *H. s. s*.

We used data on the chromosomal localization of 1595 experimentally confirmed *H. s. s*. pre-miRNAs in the miRBase database rel. 19 [11] and selected the best-sequenced *H. s. d*. and *H. s. n*. pre-miRNAs orthologous to *H. s. s*. pre-miRNA genes. The *H. s. d*. and *H. s. n*. genomes appear as short nucleotide sequences (reads) mapped onto the human genome [3]. To ensure that only the best-sequenced *H. s. d*. and *H. s. n*. pre-miRNAs would remain, we used filtering methods.

(I) The consensus sequences of *H. s. d*. and *H. s. n*. pre-miRNAs were combined from reads with a quality of mapping onto *H. s. s*. pre-miRNA genes not less than 15 [3].

(II) The consensus sequences of *H. s. d*. and *H. s. n*. pre-miRNAs included only those read nucleotides (a) for which the Phred sequence quality [25] was not less than 30 [2] and (b) that were located in the middle part of the reads: the first and last three read nucleotides were discarded according to [3].

(III) Any position in the consensus with coverage of less than 5 was assumed to be undetermined [3].

(IV) The consensus sequences of *H. s. d*. and *H. s. n*. pre-miRNAs with undetermined positions were discarded.

(V) The consensus sequences of *H. s. d*. and *H. s. n*. pre-miRNAs that (a) had nucleotide substitutions that *H. s. s*. lacked and (b) were combined from reads mapped onto the human genome with a quality of less than 30 were discarded [2,3].

(VI) If there were two or more polymorphic states at a consensus position in *H. s. d*. and *H. s. n*. pre-miRNAs, only those alternative states that (a) had comparable frequencies (the difference between the frequencies would not exceed 1.3) and (b) passed filtering stages I-V were considered.

(VII) Additionally, we manually analyzed all mapped reads of selected *H. s. d*. and *H. s. n*. pre-miRNAs for the presence of PCR artifacts. We did not find any PCR artifacts in the positions of selected *H. s. d*. and *H. s. n*. pre-miRNAs.

To select pre-miRNA coding genes that were present in the genome of the common ancestor of *H. s. s.*, *H. s. d.* and *H. s. n.* we analyzed the multiple genome alignment of six primates from Ensembl rel. 69 [12]. The minimum length of any ortholog (excluding unreadable and polymorphic nucleotides) was considered to be not less than 70% of the length of the corresponding *H. s. s*. pre-miRNA.

We mapped all the nucleotide substitutions found by comparing the pre-miRNA orthologs in the *H. s. s*./*H. s. d*. and *H. s. s*./*H. s. n*. pairs onto the secondary structures of the corresponding pre-miRNAs in *H. s. s*. contained in the miRBase database rel. 19 [11].

We selected *H. s. d*. and *H. s. n*. pre-miRNA genes with (i) nucleotide substitutions (deletions, insertions) in the regions corresponding to the sequences of mature miRNAs (and/or miRNAs^*^) or (ii) multiple substitutions within a region not larger than 1/20 of the pre-miRNA length. In the first case (i), the altered mature miRNAs are of special interest because they change the pattern of complementary interactions between miRNAs and their target mRNAs in *H. s. d*. or *H. s. n*. and make it different from *H. s. s*. In the second case (ii), the probability of observing multiple changes within a small region of *H. s. d*. or *H. s. n*. pre-miRNA by random chance is extremely low. For instance, the estimate of the total number of differences between *H. s. d*. and *H. s. s*. is approximately 1,650,000 [3]. Under the assumption of a uniform distribution of mutation fixation events in a 6400 bp long sequence (1/20 of the length of 1600 pre-miRNA genes, each gene being approximately 80 bp in length), the estimate of the probability of two mutations being fixed simultaneously is less than 0.002.

To ensure that the mutations in our selection of miRNAs were not DNA sequencing errors, we analyzed the frequencies of all polymorphic consensuses of *H. s. d*. and *H. s. n*. DNA (see steps V and VI of the filtering protocol), considering their tetranucleotide context. Thus, we used data in the UCSC Genome Browser phyloP46way to select evolutionarily conserved regions of the human genome (phyloP conservation score > 0.1; regions were ≥7 bp in length, which eventually totaled 454775413 positions or ~15% of the human genome). The occurrence of the polymorphic variants of the consensuses in the *H. s. d*. and *H. s. n*. genomes was then assessed considering their tetranucleotide context. We selected pre-miRNAs with mutations that either do not occur in the 454,775,413-strong selection of evolutionary conservative positions analyzed (p ≤ 2·10^-9^) or are single occurrences (p = 2·10^-9^).

To ensure that the mutations in the set of selected miRNAs were not a result of PCR amplification in DNA sequencing (see step VII of the filtering protocol), we performed a manual analysis of *H. s. d*. and *H. s. n*. short reads alignment on a reference *H. s. s*. genome (hg19). We found no traces of the PCR amplification of short reads in miRNA genome positions: any single read in these positions started and stopped at unique genome locations.

Using the blastn program in BLAST 2.2.26+ [26] on the pre-miRNAs containing substitutions/polymorphisms in mature miRNAs at the last filtering stage, we discarded pre-miRNAs that occurred in more than one copy in the *H. s. s*. genome (E-value cut-off: 1·10^-6^; blastn default settings).

### Identification of genes targeted by H. s. s. miRNAs orthologous to H. s. d. and H. s. n. miRNAs

For each orthologous *H. s. s*. miRNA, we identified the genes encoding its target mRNAs. This identification was performed using miRGator 3.0 [13] (we used only those target mRNAs whose expression correlated with microRNA expression with *r*<-0.9) and the ChIP-seq data for H3K4me3 histones marking active promoters in primate cortical neurons [14]. We used the ID converter from bioDBnet [27] to convert the HUGO name list of target mRNA IDs (from miRGator 3.0 prediction) into a list of Ensembl gene IDs and a list of Refseq IDs.

For functional annotation of the mRNAs targeted by the selected miRNAs, we chose two independent sources of experimental data that contained the most complete quantitative information on mRNA expression in human central nervous system tissues. One of these sources was BrainSpan [19] (Exon microarray, 16767 genes), which integrates normalized quantitative data on gene expression provided by expression time series experiments in broad brain regions. The other source of data was the Allen Human Brain Atlas, as updated March 2013 [18] (20791 genes), which contains normalized quantitative data on the expression of mRNAs in tiny brain regions. The expression of these mRNAs was analyzed in six brains: H0351.1009, H0351.1012, H0351.1015, H0351.1016, H0351.2001 and H0351.2002. If the microarray contained more than one probe for measuring the expression of the same mRNA, then only the probe producing the maximum value that significantly differed from background values was taken for analysis. In addition, to improve the robustness of our results, we used only data that were consistent for at least 4 out of 6 brains.

### Identification of tissues with increased levels of mRNA expression regulated by the pool of miRNAs

In our work, we are interested in the tissues and brain structures most likely affected by the pool of miRNAs with structural changes between ancient and modern humans. Unfortunately, the data on miRNA expression in the vast majority of human tissues and brain structures are currently incomplete both because the number of functional miRNAs is incomplete [30] and because of the number of functional miRNA/target-mRNA interactions under ongoing debates regarding the usage of various factors in miRNA/target-mRNA binding [31,32]. Therefore, we used an indirect approach to identify tissues subject to dynamical changes in miRNA expression. Because miRNAs are negative post-transcriptional regulators of gene expression, we suppose that changes in their expression levels will largely affect the tissues with high abundance of their mRNA targets. Based on this assumption, likely candidate tissues are the ones with high expression of the target mRNAs for the set of miRNAs under consideration. To express quantitatively the degree of post-transcriptional regulation by specific miRNAs in different tissues, we introduced the miRNAs' regulatory potential.

Let *W*(*k*) denote the set of human mRNAs expressed in the *k*-th tissue and targeted by all known human miRNAs. It should be noted that the overwhelming majority of genes in the human genome (~20000) [33] can be targeted by various miRNAs [13,30,34]. Therefore, the set of all genes in the genome is a good approximation of the miRNA targetome. Thus, we approximated *W*(*k*) as the set of all human mRNAs expressed in the *k*-th tissue. *W*(*k_Q_*) denotes the subset of target mRNAs regulated in the *k*-th tissue by the miRNA pool, *Q*, and *J*(*k_Q_*) denotes the size of this subset.

The regulatory potential, $L_{k}^{Q}$, exerted by the miRNA pool, *Q*, on mRNAs with expression in the *k*-th tissue was estimated using the following formula:

(1) $$L_{k}^{Q}=\sum_{j\in W(k_{Q})}E_{j}^{k}$$

Here, $E_{j}^{k}$ is the level of expression of the *j*-th target mRNA in the subset *W*(*k_Q_*) in the *k*-th tissue, and $E_{j}^{k}$ values were obtained using data from BrainSpan [18] or AHBA[19]. The $L_{k}^{Q}$ value obtained in this manner corresponds to the number of mRNA molecules that can be regulated in the *k*-th tissue (or brain structure) by mature miRNAs in the pool *Q *and characterizes its post-transcriptional regulatory potential in that tissue.

**Figure 5:**
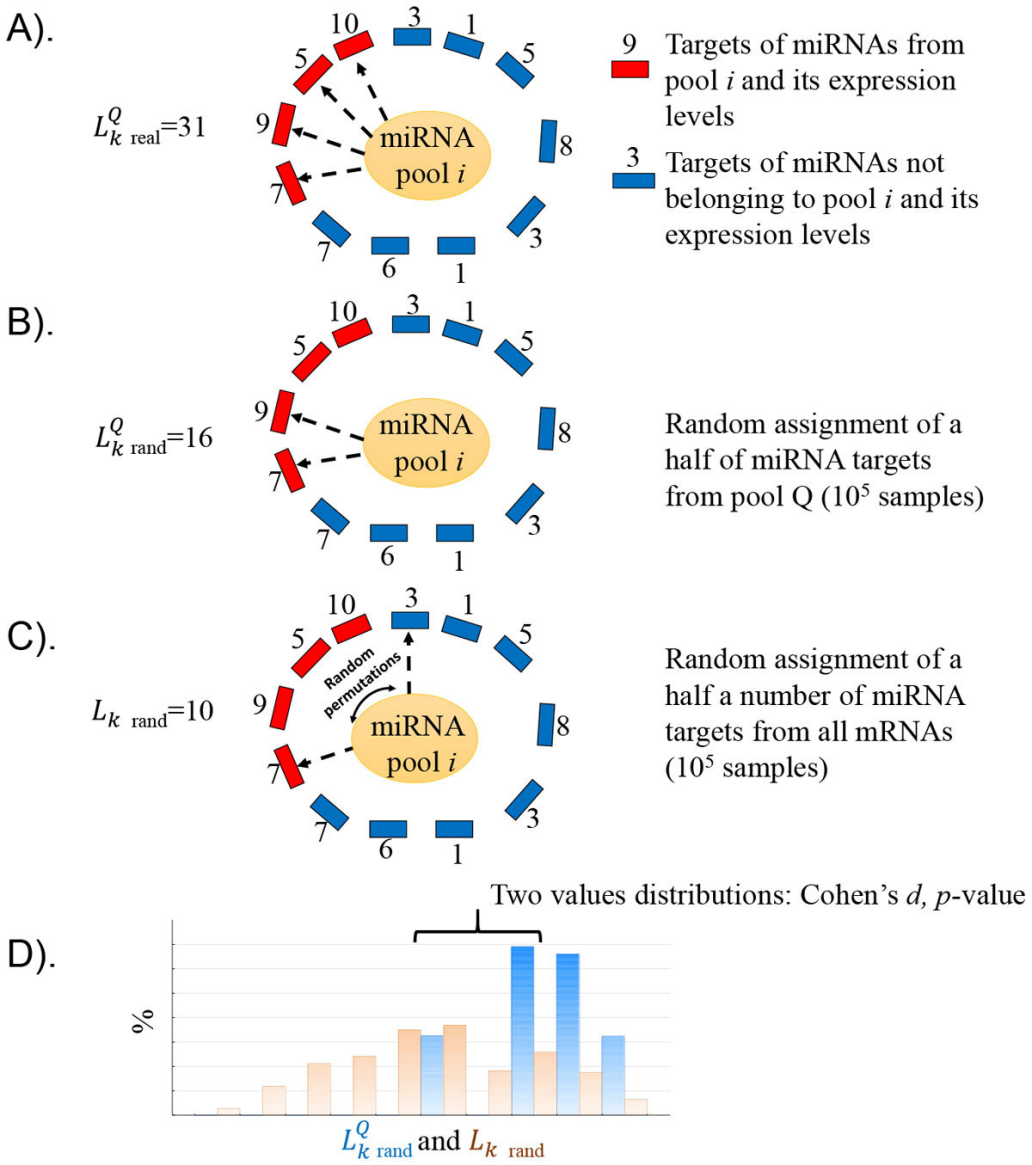
**A toy example of the resampling test to identify tissues with increased levels of mRNAs expression regulated by the pool *Q *of the miRNAs in tissue *k***. A). Calculation of the regulatory potential for the miRNA pool *Q*, $L_{k}^{Q}$. B). Calculation of the regulatory potential $L_{krand}^{Q}$ for the resampled miRNA pool *Q *in *W*(*k_Qrand_*) subset. C). Calculation of the regulatory potential *L_krand _*in the *W*(*k_rand_*) subset. D). Distribution of $L_{krand}^{Q}$ (blue) and *L_krand _*(brown) values in 10^5^ resampling experiments and their comparison by Cohen's *d *statistics.

In search of the tissues targeted by the miRNAs from the pool *Q*, we selected the ones with the highest regulatory potential $L_{k}^{Q}$ in comparison with the background level of expression of all miRNA targets expressed in the *k*-th tissue, *W*(*k*). To estimate the significance of the difference between the pool's *Q *regulatory potential and the background, we used a resampling test (see Figure 5).

First, we performed random sampling without replacement of *J*(*k_Q_*)/2 mRNAs from the set *W*(*k_Q_*) of human mRNAs targeted by miRNAs in the pool *Q *(the *W*(*k_Qrand_*) subset). For the *W*(*k_Qrand_*) subset, we calculated $L_{krand}^{Q}$ using Formula (1). The sampling procedure was repeated 10^5 ^times. We estimated the mean and standard deviation of the $L_{krand}^{Q}$ values. The selection of half the number of mRNAs in the *W*(*k_Q_*) subset allowed us to account for uncertainties in miRNA annotation (for example, the possibility that the miRNA dataset under consideration is incomplete and has incomplete annotation of target mRNAs).

Second, we performed sampling of mRNAs from the background distribution, using the *W*(*k*) set of all miRNA targets expressed in the *k*-th tissue. For this purpose, we selected *J*(*k_Q_*)/2 mRNAs from the *W*(*k*) randomly without replacement. We termed this subset *W*(*k_rand_*). We calculated

(2) $$L_{krand}=\sum_{j\in W(k_{rand})}E_{j}^{k},$$

as the estimate of the background miRNA regulatory potential; here, $E_{j}^{k}$ is the expression level of the *j*-th target mRNA in the subset *W*(*k_rand_*). This procedure was repeated 10^5 ^times and allowed us to estimate the mean and standard deviation of *L_krand_*.

We calculated the difference between the *L_krand _*and $L_{krand}^{Q}$ value distributions using Cohen's *d *statistic (effect size value) [35,36]. The *p*-value of $L_{krand}\geq L_{krand}^{Q}$ was calculated using Student's *t*-statistic. The difference between the *L_krand _*and $L_{krand}^{Q}$ value distributions was considered significant at *p*-value less or equal to 0.01, implying that the *k*-th tissue was targeted by the miRNAs from the pool *Q*. It is important to note that the data resampling approach described above does not rely on distributional assumptions and simultaneously allowed us to control Type I error, and corrects for any hidden data correlation [37-39]. Therefore, we did not need to perform multiple testing corrections (e.g., Bonferroni correction) of the significance level [36-39] and therefore could use the selected *p*-value threshold directly.

## Competing interests

The authors have declared that no competing interests exist.

## Authors' contributions

K.V.G., A.D.A. and E.I.R. conceived the project. K.V.G. performed all data analysis. N.A.K., E.I.R. and A.P.D. coordinated the project. All authors contributed to the final manuscript preparation, discussed the results and their implications, and have read and approved the final manuscript.
